# Factors Associated with Recurrence of Varicose Veins after Thermal Ablation: Results of The Recurrent Veins after Thermal Ablation Study

**DOI:** 10.1155/2014/505843

**Published:** 2014-01-27

**Authors:** R. G. Bush, P. Bush, J. Flanagan, R. Fritz, T. Gueldner, J. Koziarski, K. McMullen, G. Zumbro

**Affiliations:** ^1^Midwest Vein & Laser Center, Centerville, OH 45459, USA; ^2^Delaware Valley Vein Center, Phoenixville, PA 19460, USA; ^3^Advanced Vein Center of North Texas, Irving, TX 75038, USA; ^4^Wisconsin Vein Center, Manitowoc, WI 54220, USA; ^5^Family Surgical, Battle Creek, MI 49015, USA; ^6^Varicose Vein Clinics of Oklahoma, Oklahoma City, OK 73120, USA; ^7^Vein Specialists of Augusta, Martinez, GA 30907, USA

## Abstract

*Background*. The goal of this retrospective cohort study (REVATA) was to determine the site, source, and contributory factors of varicose vein recurrence after radiofrequency (RF) and laser ablation. *Methods*. Seven centers enrolled patients into the study over a 1-year period. All patients underwent previous thermal ablation of the great saphenous vein (GSV), small saphenous vein (SSV), or anterior accessory great saphenous vein (AAGSV). From a specific designed study tool, the etiology of recurrence was identified. *Results*. 2,380 patients were evaluated during this time frame. A total of 164 patients had varicose vein recurrence at a median of 3 years. GSV ablation was the initial treatment in 159 patients (RF: 33, laser: 126, 52 of these patients had either SSV or AAGSV ablation concurrently). Total or partial GSV recanalization occurred in 47 patients. New AAGSV reflux occurred in 40 patients, and new SSV reflux occurred in 24 patients. Perforator pathology was present in 64% of patients. *Conclusion*. Recurrence of varicose veins occurred at a median of 3 years after procedure. The four most important factors associated with recurrent veins included perforating veins, recanalized GSV, new AAGSV reflux, and new SSV reflux in decreasing frequency. Patients who underwent RF treatment had a statistically higher rate of recanalization than those treated with laser.

## 1. Introduction

Recurrent varicose veins are known to be a common problem after surgery, Recurrent veins after surgery (REVAS), in patients with chronic venous disease. The incidence of those patients with REVAS is reported to be between 20% and 80% [1–4]. In 2006, a study was designed to identify patients with REVAS according to CEAP classification as well as the source, site, and cause of recurrence. The results of the REVAS study revealed recurrent venous disease to be associated with perforators, neovascularity, and recurrent saphenous insufficiency from multiple etiologies [5].

Thermal ablation of the saphenous vein has been performed for over 10 years with yearly increases in frequency. While published studies have verified 95% successful ablations at 3–5 years [6, 7], detailed analysis of recurrent venous disease after thermal ablation (REVATA) is minimal. The REVATA study was designed to identify those patients with recurrent symptomatic venous disease after thermal ablation with either laser or radiofrequency (RF), presenting with new varices and symptoms in a period ranging up to 8 years after the initial procedure.

## 2. Methods

Seven centers were selected for this retrospective cohort study. Criteria for selection included the following. (1) All participating surgeons were board certified in general or vascular surgery. (2) All participants had at least a five-year experience in the performance of thermal ablation. (3) Each participant performed at least 100 cases of thermal ablations per year. The data collection study tool developed for this investigation was uniquely designed for this study.

In 2010, 2,380 patients were seen at 7 participating centers for evaluation of symptomatic venous disease. Of the total patients seen, 164 were identified as having recurrent venous disease after a previous thermal ablation and these patients form the basis of this study. Of the 164 patients, 83% of this number had their initial treatment by the participating surgeons in this study also, reevaluating and performing the second procedure in the designated study time frame of one year. The number of patients evaluated and treated by the 7 participating surgeons in the REVATA study ranged from 10 to 35 for the one-year time frame. Each surgeon completed the data collection study tool on each qualified patient. After completing the study tool, the form was reviewed for accuracy and resulting data was given a numerical value. The data was entered into the Statistical Package for the Social Science (SPSS) for statistical analysis.

Data was entered into 15 categories for each patient in the study. Gender, age, initial procedure, and time to recurrence were documented. At time of presentation for recurrent venous disease, careful documentation of ultrasound (US) findings was recorded. The patients were examined in the standing position and reflux >0.5 seconds was considered positive. The US findings of the greater saphenous vein (GSV) were entered in 1 of 8 categories (Table 1). Duplex US findings of the small saphenous vein (SSV) and anterior accessory saphenous vein (AAGSV) were entered in 1 of 4 categories (Tables 2 and 3). Documentation of perforators with or without associated truncal vein reflux was noted. The perforators were categorized as to thigh or calf without further localization.

The deep venous system was evaluated by examination of the femoral and popliteal vein. The technique used to evaluate the deep veins was calf compression. Retrograde flow of >1 second was considered positive for reflux. Notation was also made of prior history of deep vein thrombosis (DVT).

The original mechanism of thermal ablation, laser, or RF was noted. Laser ablation was further subdivided into 4 categories depending on the laser utilized. In addition, each participating surgeon entered if known the average joules/cm used during the laser ablation. The procedure performed in 2010 for treatment of recurrent venous disease was documented. In the majority of cases, foam injection was used for incompetent perforators.

## 3. Results

Of the 164 patients entered into the study, 83% was female and 17% was males. The mean age was 53. The initial procedure was GSV ablation with or without phlebectomy in 97% of patients. Two patients had as their initial procedure, SSV ablation and 3 patients had AAGSV ablation as their primary procedure. Forty-three patients had concurrent ablation of the SSV (9 RF, 34 laser). Phlebectomies were routinely performed in conjunction with thermal ablation if varices were present. Treatment of perforators was not documented in any patient at the time of original ablation.

The mechanism of original ablation was RF in 33 patients and laser in 131 patients. Laser ablation was subdivided into type of laser utilized with over 50% performed with the 940 nm wavelength. The median time to recurrence of symptomatic varicose veins was 3 years. The presenting clinical findings are listed in Table 4. Of the recurrences, 47 patients (29%) had either partial or total recanalization of the GSV. Twenty-three patients (14%) had new saphenous insufficiency in previously unablated GSV segment. For those patients with recanalization, the etiology was either branch or perforator inflow. New reflux in the AAGSV occurred in 40 patients (24%) of the 164 in this study undergoing prior GSV ablation. New SSV insufficiency occurred in 27 patients (16%).

The presence of perforators in those patients with recurrent disease was demonstrated in 126 patients (77%). Saphenous recanalization in conjunction with perforators was documented in 17 patients. New saphenous insufficiency into an untreated below knee GSV occurred in 23 patients. In this group of 23 patients, the insufficiency was secondary to either a branch or perforator in direct continuity with the GSV segment. Perforators were associated with 4 cases of recanalized SSV. New reflux in the SSV was associated with perforators in 19 patients. These perforators were in the medial calf area for the most part and were not in continuity with the SSV. Of the total of 126 patients with documented perforators, 64% had recurrent disease associated with a pathologic perforator. The presenting clinical picture included truncal vein insufficiency, recanalization, isolated varices, stasis, or ulcer formation.

A comparison was completed between the use of RF and lasers and the resultant rate of recanalization. In addition a comparison was also completed with the different laser wavelengths and the success of thermal ablation. Only the 940 nm, 810 nm, and 1320 nm lasers had sufficient numbers for statistical analysis (Table 5).

A two-way contingency table analysis was conducted to assess whether the use of RF as the mechanism for the original ablation led to greater frequencies of GSV saphenous recanalization. Mechanism used for the original ablation and GSV recanalization was found to be significantly related, Pearson *χ* ^2^ (4, *N* = 139) = 69.79, *P* < .001, and Cramer's *V* = .71. The proportions of GSV recanalization across RF, laser 940 nm and laser 810 nm were .90, .13, .44, respectively. Laser 1320 nm had no instances of recanalization.

Follow-up pairwise comparisons were conducted to evaluate the difference among the proportions previously listed. A significant difference was found between use of RF and all lasers. Recanalization probability was 7.2 times (.90/.13) more likely when the mechanism used to treat the original ablation was RF as opposed to laser 940. Comparing RF to 810 nm laser, the risk of recanalization was about 2 times more likely when RF was used. The probability of the GSV being ablated was about 10 times more likely when the mechanism used to treat the original ablation was laser 1320 as opposed to RF. Comparing lasers demonstrated a significant difference between the 940 nm and the 1320 nm laser compared to the 810 nm laser.

The presence of perforators in those patients with recurrent disease was demonstrated in 126 patients (77%). Saphenous recanalization in conjunction with perforators was documented in 17 patients. A two-way contingency table analysis found presence of perforators to be significantly associated with GSV recanalization, Pearson *χ* ^2^ (3, *N* = 139) = 10/95, *P* < .05, and Cramer's *V* = .28. The proportions of GSV recanalization across thigh, calf, and both thigh and calf were .70, .23, and .32, respectively.

Follow-up pairwise comparisons were conducted to evaluate the difference among these proportions. The only significant difference was found between varicosities associated with perforator in the thigh and varicosities associated with perforator in the calf. The probability of a recanalization occurring was about 3.04 times (.70/.23) more likely when varicosities associated with perforator in the thigh occurred as opposed to that in the calf.

## 4. Discussion

Recurrent venous disease after surgery has been well documented. In many of the cases, neovascularity or technical failures have contributed to GSV insufficiency and subsequent recurrent varicose vein formation. The majority of patients also had the presence of perforating veins documented as well.

In the REVATA study, a significant difference in the proportion of patients with neovascularity was evident. A significantly greater proportion of patients have neovascularity occurring after surgery, when compared to the REVATA study. An interesting finding is that neovascularity in the REVATA study rarely occurred in the region of the SFJ, but more distal. In 40% of cases, an incompetent perforator was documented adjacent to the neovascularity. The lower incidence of neovascularity would be expected since there was not a crossectomy performed. One of the reasons for neovascularity has been postulated to be the result of an angiogenic response to surgical trauma [1, 8]. The incidence of neovascularity in thermally ablated areas, although lower, suggests that heat injury can induce this phenomenon.

The REVATA study confirmed a significant difference between the use of RF and laser in GSV recanalization. The results showed that greater proportions of GSV recanalization occurred when RF was used as the instrument for the original ablation as opposed to laser.

The REVATA study is simply a comparison of different modalities in the study group. The authors do not know the number (denominator) of all patients treated prior to this study. The study demonstrates only the statistical probability of the etiology of reoccurrence of the different treatment modalities in the 164 patients (numerator) only. A limitation to the study is that expected counts of less than 5 were present in 6 of the 8 significant comparisons in this cross-sectional study.

In the REVATA RF group, the majority of patients, but not all, were treated with the older generation RF devices. Further studies and longer follow-up may demonstrate that RF outcomes would improve with the new model of RF ablations.

Follow-up pairwise comparisons also found significant differences between types of laser and GSV recanalization. The energy in the 940 nm group averaged 80 joules/cm. The average energy in the 1320 nm group was 100 joules/cm. The laser with the most failures was the 810 nm wavelength. In this group, 3 of the thermal ablation failures occurred in patients with less than 60 joules/cm of energy. Decreased energy application has been reported to be a factor in the failure of thermal ablation [9, 10].

A significant difference was found between the presence of thigh perforators and GSV recanalization. Specifically, GSV recanalization had a greater proportion of occurrence when there was an associated thigh perforator than when there was an isolated calf perforator. Perforators of the calf were associated with a higher rate of new GSV insufficiency. The higher proportion of GSV recanalization with thigh perforators documented in this study may be the result of the midcalf level saphenous vein not being treated initially. Future studies can easily determine the true proportional significance between the thigh and calf perforator recanalization rates.

Additionally, the etiology of thigh perforators in ablation failure may be the result of “arterialization.” The authors have noted instances of a localized arterial venous flow pattern following thermal ablation in the region of the perforators. Usually the flow pattern, if it occurs, is within the first 6 months after thermal ablation. This phenomenon may be related to thermal injury, since the US findings are in close proximity to the thermally treated saphenous vein. Flow with increased pressure would result making recanalization possible. Femoral vein reflux could possibly elevate the venous pressure making recanalization possible, but this factor has not been documented. Additionally in the REVATA study, the presence of femoral deep venous insufficiency (DVI) was not associated with an increased recanalization rate, although the presence of DVI has been documented as an etiology of recurrence.

As much as possible, the authors recommend the following protocol for repeat US after thermal ablation: 2-3 days, 1 month, 6 months, and 1 year. This protocol has allowed the early identification of persistent or new refluxing perforating veins. If identified, treatment with US guided foam sclerotherapy (1%) is done. Recanalization after thermal ablation is now exceedingly rare with close follow-up.

New saphenous insufficiency in unablated segments of the GSV occurred in 23 patients. In this group of 23 patients, the resulting flow was secondary to either a branch or perforator in direct continuity with the GSV segment. The main perforators responsible for recurrent insufficiency in nonablative segments are the paratibial perforators, which correspond to Sherman's and Boyd's in the old nomenclature. If possible, ablation should begin at midcalf level below these perforators to reduce the chance of future new insufficiency in untreated segments.

Perforators were associated with 4 cases of recanalized SSV. New reflux in the SSV was associated with perforators in 19 patients. These perforators were in the medial calf area for the most part and were not in continuity with the SSV. Of the total of 136 patients with documented perforators, 64% had recurrent disease associated with a pathologic perforator. The presenting clinical picture included axial vein insufficiency, recanalization, isolated varices, stasis, or ulcer formation. The majority of the pathologic perforators were treated with foam injection.

Forty-five thermal ablations of the SSV were performed as either an initial or secondary ablation at the time of the original treatment. The number of successful ablations with no recanalizations was 88%.

Many reports have documented a high recurrence rate after ligation and/or stripping of the SSV. Recurrence rates as high as 49% have been described [11]. Labropoulos et al. [12] have determined that SSV reflux was responsible for 29% of recurrent varices in the REVAS group. Most of the recurrences of SSV insufficiency have been attributed to inadequate (SPJ) dissection. Unligated branches, anatomical variations, and retained SSV have been commonly cited as an etiology [11].

While this subgroup is not a retrospective study of all SSVs performed, the high percentage of successful SSV ablations is in marked contrast to results of previous reports using standard surgical techniques. Technical and tactical failures should be nonexistent using standard protocol with thermal ablation. For the subgroup of patients in the REVATA study, the successful SSV ablations are equivalent to that reported by Ravi et al. [13].

In the REVATA study, 16% of patients had new SSV insufficiency. All these patients had a previously documented nonrefluxing SSV on their initial US prior to 2010. Patients with new SSV insufficiency were treated with thermal ablation and the majorities were treated with the laser.

New AAGSV insufficiency occurred in 40 patients (24%) after the original procedure of GSV ablation. The high number of patients developing AAGSV insufficiency in the follow-up in this series is important to be noted. One possible explanation is that once the GSV is ablated, flow is then directed to the AAGSV. Due to inherent defects in vein wall or valves, resultant insufficiency occurs. Prior to GSV ablation, refluxing flow preferentially follows the larger diameter GSV. Of interest is that the percentage of patients with new AAGSV insufficiency parallels that of the number of patients who developed neovascularity at the groin level in the REVAS study [5].

A true percentage of all recurrent veins after thermal ablation cannot be determined due to the study design and parameters. Future studies can be designed to answer this question. However, with the rate of recurrence after surgery at 2 years being in the range of 25%–30% [1] and higher at 5 years [14, 15], the low rates of recurrences after thermal ablations seen in this large study group may prove to be statistically significant.

## 5. Conclusion

The majority of recurrences were in association with perforating veins. New AAGSV and SSV insufficiency was responsible for 40% of those patients who developed recurrent venous disease. The use of standard protocols and routine US examinations may reduce the frequency of saphenous vein recanalization after thermal ablation. New insufficiency in the unablated GSV can be reduced by beginning thermal ablations at midcalf. In the REVATA study, laser ablation of the GSV was statistically superior to RF, using first generation devices.

## Figures and Tables

**Table 1:** Repeat US GSV.

	Frequency	Percent	Valid percent	Cumulative percent
Saphenous recanalization total	13	7.9	7.9	7.9
Saphenous recanalization partial	7	4.3	4.3	12.2
Saphenous recanalization associated with perforators	17	10.4	10.4	22.6
Saphenous recanalization associated with branch inflow	10	6.1	6.1	28.7
Saphenous insufficiency in unablated segments	13	7.9	7.9	36.6
Saphenous insufficiency in unablated segments associated with perforator	10	6.1	6.1	42.7
Ablated	92	56.1	56.1	98.8
Normal	2	1.2	1.2	100.0

Total	164	100.0	100.0	

**Table 2:** Repeat US SSV.

	Frequency	Percent	Valid percent	Cumulative percent
Recanalized SSV total	5	3.0	3.0	3.0
New reflux SSV	27	16.5	16.5	19.5
Ablated	40	24.4	24.4	43.9
Normal	92	56.1	56.1	100.0

Total	164	100.0	100.0	

**Table 3:** Repeat US AAGSV.

	Frequency	Percent	Valid percent	Cumulative percent
Recanalized AAGSV total	2	1.2	1.2	1.2
Recanalized AAGSV partial	2	1.2	1.2	2.4
New reflux AAGSV	40	24.4	24.4	26.8
Ablated	6	3.7	3.7	30.5
Normal	114	69.5	69.5	100.0

Total	164	100.0	100.0	

**Table 4:** CEAP class at 2010 evaluation.

	Frequency	Percent	Valid Percent	Cumulative Percent
Visible varicose veins may have symptoms	75	45.7	45.7	45.7
Swelling and/or edema	77	47.0	47.0	92.7
Skin changes such as stasis dermatitis	2	1.2	1.2	93.9
Healed ulcer	1	0.6	0.6	94.5
Ulcer present	9	5.5	5.5	100.0

Total	164	100.0	100.0	

**Table 5:** original ablation ∗ repeat US GSV crosstabulation.

	Repeat US GSV								Total
	Saphenous recanalization total	Saphenous recanalization partial	Saphenous recanalization associated with perforators	Saphenous recanalization associated with branch inflow	Saphenous insufficiency in unablated segments	Saphenous insufficiency in unablated segments associated with a perforator	Ablated	Normal	
*Original ablation *									
RF									
Count	5	4	13	5	1	2	3	0	33
% within original ablation	15.2%	12.1%	39.4%	15.2%	3.0%	6.1%	9.1%	0.0%	100.0%
Laser 940									
Count	0	1	4	2	11	8	49	1	76
% within original ablation	0.0%	1.3%	5.3%	2.6%	14.5%	10.5%	64.5%	1.3%	100.0%
Laser 810									
Count	5	1	0	1	0	0	9	1	17
% within original ablation	29.4%	5.9%	0.0%	5.9%	0.0%	0.0%	52.9%	5.9%	100.0%
Laser 1470									
Count	1	0	0	0	0	0	0	0	1
% within original ablation	100.0%	0.0%	0.0%	0.0%	0.0%	0.0%	0.0%	0.0%	100.0%
Laser 920									
Count	2	1	0	2	1	0	5	0	11
% within original ablation	18.2%	9.1%	0.0%	18.2%	9.1%	0.0%	45.5%	0.0%	100.0%
Laser 1320									
Count	0	0	0	0	0	0	26	0	26
% within original ablation	0.0%	0.0%	0.0%	0.0%	0.0%	0.0%	100.0%	0.0%	100.0%

Total									
Count	13	7	17	10	13	10	92	2	164
% within original ablation	7.9%	4.3%	10.4%	6.1%	7.9%	6.1%	56.1%	1.2%	100.0%
